# Understanding porosity and temperature induced variabilities in interface, mechanical characteristics and thermal conductivity of borophene membranes

**DOI:** 10.1038/s41598-021-91705-2

**Published:** 2021-06-09

**Authors:** Van-Trung Pham, Te-Hua Fang

**Affiliations:** 1 Department of Mechanical Engineering, National Kaohsiung University of Science and Technology, Kaohsiung, 807 Taiwan; 2 Department of Mechanical Engineering, Pham Van Dong University, Quang Ngai, 570000 Vietnam

**Keywords:** Nanoscience and technology, Materials science, Nanoscale materials, Theory and computation, Atomic and molecular physics

## Abstract

Evaluating the effect of porosity and ambient temperature on mechanical characteristics and thermal conductivity is vital for practical application and fundamental material property. Here we report that ambient temperature and porosity greatly influence fracture behavior and material properties. With the existence of the pore, the most significant stresses will be concentrated around the pore position during the uniaxial and biaxial processes, making fracture easier to occur than when tensing the perfect sheet. Ultimate strength and Young’s modulus degrade as porosity increases. The ultimate strength and Young's modulus in the zigzag direction is lower than the armchair one, proving that the borophene membrane has anisotropy characteristics. The deformation behavior of borophene sheets when stretching biaxial is more complicated and rough than that of uniaxial tension. In addition, the results show that the ultimate strength, failure strain, and Young’s modulus degrade with growing temperature. Besides the tensile test, this paper also uses the non-equilibrium molecular dynamics (NEMD) approach to investigate the effects of length size, porosity, and temperature on the thermal conductivity (*κ*) of borophene membranes. The result points out that *κ* increases as the length increases. As the ambient temperature increases, *κ* decreases. Interestingly, the more porosity increases, the more *κ* decreases. Moreover, the results also show that the borophene membrane is anisotropic in heat transfer.

## Introduction

Since the successful synthesis of graphene in 2004^1^, two-dimensional (2D) nanomaterials have attracted much attention due to their novel mechanical, thermal, electronic, optical properties. For example, many other 2D nanomaterials, such as silicene, MoS_2_, hBN germanene, and phosphorene, have been studied^2–8^. Recently, the 2D boron membrane, named borophene material, was successfully synthesized for the first time in 2015^9^. This type of material strongly attracts researchers due to its unique properties. Borophene sheet possesses a negative Poisson’s ratio and anisotropic characteristics in mechanical properties and heat transfer^5,10,11^. Therefore, this material is intensively investigated in mechanical properties, optical properties, electronic structure^12–16^. Besides, the applications of borophene materials have also been studied. For example, Liao et al.^17^ studied the applicability of borophene as energy storage materials. Adamska et al.^18^ presented an extensive first-principles study of borophene’s structural and optoelectronic characteristics. In addition, borophene has been studied for application in optical circuits, on electronic devices, or in biomedical sensors^19–23^.

When synthesizing borophene, we can not avoid defects, and they significantly affect the mechanical and thermal conductivity of the material. Some previous studies investigated the influence of defects on the mechanical characteristics of borophene membranes, while others focus on its lattice thermal conductivity. For example, our prior study of borophene material investigated the effect of the crack on the mechanical properties^24^. The effect of temperature and point defects on thermal transport characterization of borophene is investigated by Noroozi et al.^25^. Abadi et al.^26^ studied mode I fracture and the crack propagation behavior of polycrystalline borophene sheets. The effect of circular pores on thermo-mechanical properties of borophene sheets is studied using the MD simulation method via the ReaxFF potential function by Arabha et al.^27^. Some authors reported the thermal conductivities of single and multi-layer borophene and the anomalous strain effect on its thermal conductivity^28,29^. Recently machine learning interatomic potentials have been employed to examine the lattice thermal conductivity of borophene and its heterostructure with graphene^30^. However, the influence of pores uniformly distributed on the membrane and the effect of temperature and tensile strain rate have rarely been studied. In particular, the impact of temperature and porosity on the biaxial deformation process has not been studied. Therefore, studying these characteristics of borophene is essential and needs further investigation.

Most of the researches above were performed by the experimental method or first-principle calculation. Experimental studies are often done at the macro level. This method is challenging to perform at the nanoscale and has high implementation costs. While the first-principle is precise, however, since this method requires a high computation, so it is limited to small construction. An alternative method is molecular dynamics (MD) simulation, which can solve the above problems^31–34^. Besides, the MD simulations become an effective tool to analyze the mechanical behavior, heat transfer properties with the effects of various defects at the nanoscale^35–37^.

Motivated from the above analysis, in this study, we utilize MD simulations to understand the deformation behavior and mechanical characteristics of borophene membranes with various porosities and temperatures. The deformation behaviors, ultimate strength, Young’s modulus were analyzed and calculated during uniaxial and biaxial tensile processes. In addition, the non-equilibrium molecular dynamics (NEMD) approach was used to analyze the effects of borophene membrane length, porosity, and temperature on the thermal conductivity (*κ*) of the borophene membranes.

## Results

**Figure 1:**
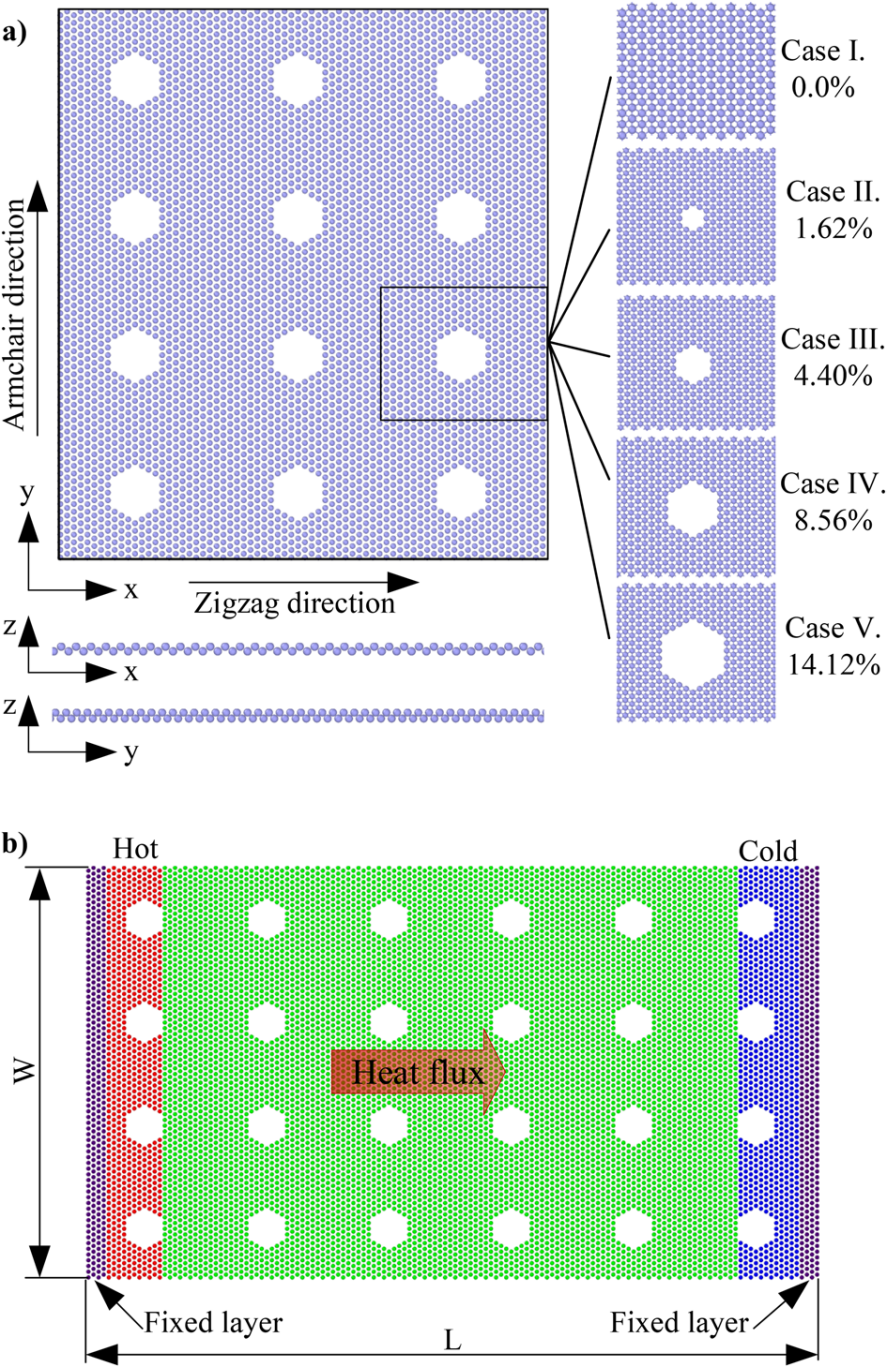
The schematic illustration of borophene membrane with different porosities (**a**) used for the uniaxial and biaxial tensile test, (**b**) used for the estimation of the thermal conductivity of borophene membrane.

To study the effect of porosity on the mechanical properties, a model of borophene membrane with different porosities was used for the uniaxial and biaxial tensile process, as shown in Fig. 1a. The pores are uniformly distributed on the nanosheet. The porosity is determined by the ratio of the missing atoms to the total number of atoms in the initial borophene nanosheet. The borophene nanosheets with different porosities of 0.0% (perfect sheet), 1.62%, 4.40%, 8.56%, and 14.12% are used in the study. The dimensions of the borophene membrane are 10.3 × 11.6 nm^2^ along the *x* and *y* directions, respectively.

To evaluate the effect of porosity and temperature on the thermal conductivity of the borophene membrane, we use a model as shown in Fig. 1b. This model has dimensions *L *×* W*, with *L* is the dimension of the heat transfer direction, *W* denotes the width of the membrane. The investigated borophene membranes also have the same porosity as shown in the model in Fig. 1a. The periodic boundary condition is employed according to the width of the membrane to avoid the boundary effect of the width dimension on the calculation result^38^. Whereas in the heat transfer (*L*) direction, the fixed boundary condition applied in the NEMD method will provide a higher computation efficiency than the periodic boundary condition, as demonstrated in Ref.^39^. So the selected model has width *W* = 10.3 nm, while the length of the membrane (*L*) is changed with different values, including 10.3, 20.6, 30.9, 40.1, and 60.7 nm to survey the effect of sizes on thermal conductivity.

### Influence of porosity on mechanical characteristics of borophene membrane in uniaxial tension test

**Figure 2:**
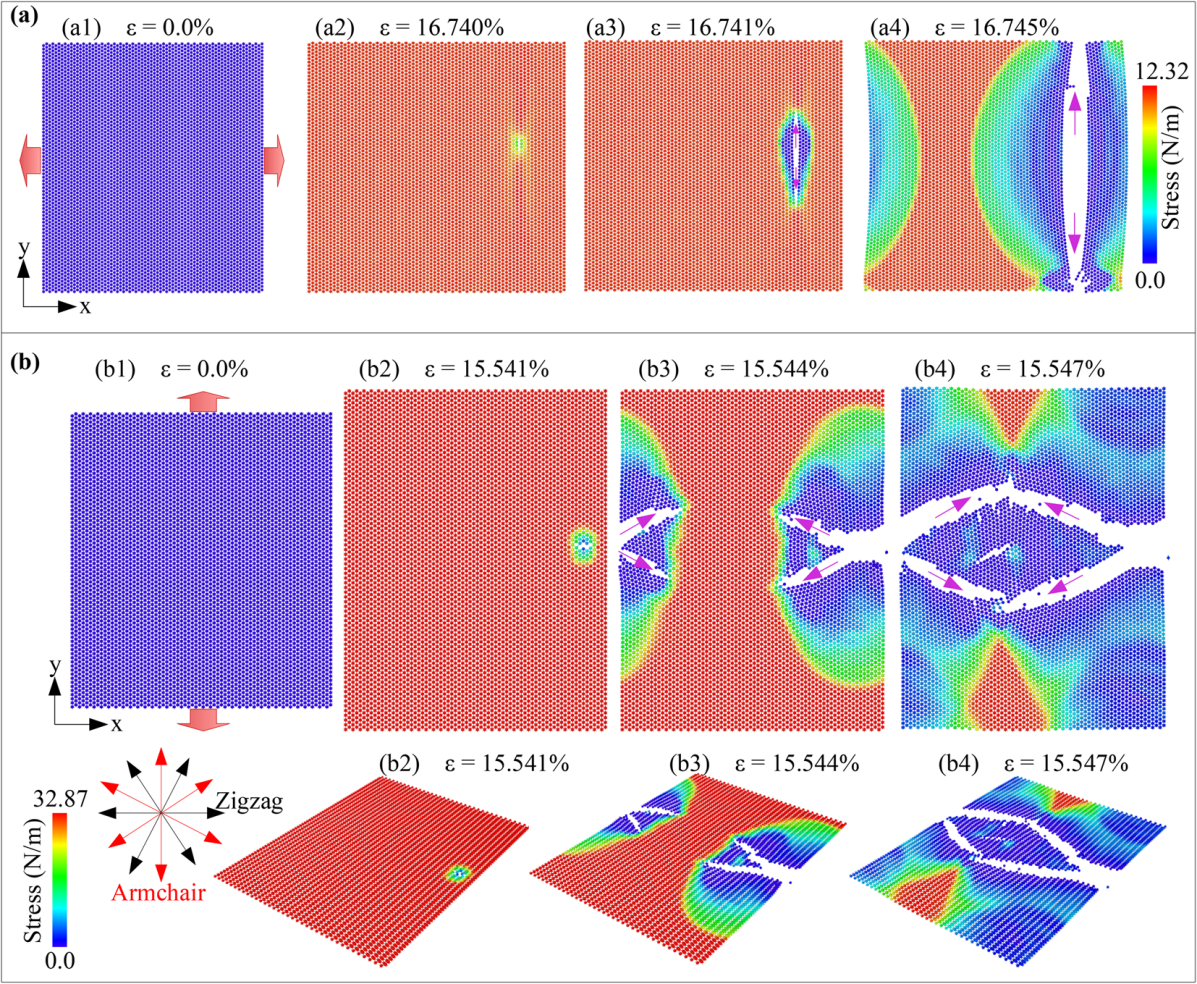
Structure evolution and fracture behaviour of monolayer borophene during uniaxial tension at 1 K (**a**) in the *x* (zigzag) direction and (**b**) in the *y* (armchair) direction.

Figure 2a shows the structural evolution and fracture process of monolayer borophene in uniaxial tension at 1 K along the *x* (zigzag) direction. All atoms are colored based on the atomic stress tensor *σ*_*xx*_. Under the stretching process in the *x-*direction, as the strain values increase, the *σ*_*xx*_ value increases. As strain rises from 0 to 16.740%, the stress in the borophene membrane rises from 0.0 to 12.32 N/m, respectively. When the strain reaches a value of 16.740%, a crack will begin to appear in the sheet. As the strain value continues to increase, the crack will rapidly develop in the direction perpendicular to the tension direction, as shown by the pink arrow. The borophene membrane is complete destruction when the strain reaches a value of 16.745%. Figure 2b presents the structural evolution of monolayer borophene in armchair tension at 1 K. All atoms are colored based on the atomic stress tensor *σ*_*yy*_. Under *y-*direction tension, when strain rises from 0.0 to 15.541%, the stress value grows from 0.0 to 32.87 N/m. At *ε* = 15.541%, the borophene membrane started to appear crack. These cracks spread in a direction that creates an angle of 60° between the tension and the crack directions. These cracks grow rapidly, and the borophene membrane is wholly destroyed at *ε* = 15.547%. Interestingly, the cracks quickly spread in the direction with the pink arrow, which is also the armchair direction. This suggests that the edge energy in the armchair edge is lower than the zigzag edge, then that the fracture deformation prefers to spread in this direction.

With various porosities of 1.62%, 4.40%, 8.56%, and 14.12%, the structural evolution and fracture process of monolayer borophene at 1.0 K in zigzag tension are shown in Supplementary Fig. S1. The results revealed that as the strain increases, the greatest stresses would be concentrated at the atoms around the pore site. As increasing deformation of nanosheets with the porosity of 1.62%, 4.40%, 8.56%, 14.12%, cracks will begin to appear at *ε* = 8.807%, 7.957%, 7.644%, and 7.511%, respectively. Like tensing the perfect membrane in a zigzag direction, these cracks also spread rapidly in a direction perpendicular to the tension direction. The results also show that as the porosity value increases, the ultimate strength and ultimate strain decrease.

In armchair tension at 1 K with different porosities of 1.62%, 4.40%, 8.56%, and 14.12%, the structural evolution and fracture process of monolayer borophene are shown in Supplementary Fig. S2. Like tensile in the zigzag direction, atoms with high-stress values will concentrate around the pores during the tension process, making the bonds of the atoms here easily destroyed first. Corresponding to the porosity of 1.62%, 4.40%, 8.56%, and 14.12%, cracks begin to appear when strain reaches values of 8.363%, 8.443%, 8.871%, and 9.348%, respectively. The crack propagation of the nanoporous sheets is different from the perfect borophene sheet. In the nanoporous sheets, the cracks propagate in a direction perpendicular to the direction of tension. This phenomenon is consistent with previous research^24^.

**Figure 3:**
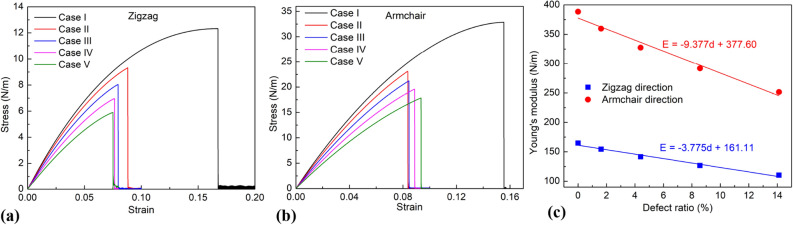
Mechanical characteristics of the borophene membrane with various porosities. (**a**) Stress–strain relationship of the borophene membranes in zigzag tension. (**b**) Stress–strain relationship of the borophene membranes in armchair tension. (**c**) Young’s moduli of the borophene membranes in the zigzag and armchair direction under uniaxial tension.

Figure 3 displays the stress–strain curves of the borophene membrane with various porosities in the zigzag direction (a), in the armchair direction (b), and Young’s moduli in the zigzag and armchair directions under uniaxial tension are shown in Fig. 3c. The pores have a strong influence on the strength of the material. The missing of B atoms disrupts the topology of the perfect borophene sheet, degrading the ultimate strength and Young's modulus. As the number of defect atoms increases (pore size increase), tensile strength and Young's modulus degrade. The Young modulus values correspond to the porosity of 0% (perfect model), 1.62%, 4.4%, 8.56%, and 14.12% respectively 164.73, 154.24, 141.46, 126.61, and 110.16 in the zigzag direction and 388.33, 359.74, 327.36, 291.79, and 251.64 according to the armchair direction. Young's modulus can be fitted into a linear relationship with porosity:

1 $$ {\text{For armchair direction}}:E = - \;9.377d + 377.60, $$

2 $$ {\text{For zigzag direction}}:E = - \;3.775d + 161.11, $$

where *E* and *d* denote Young’s modulus and porosity, respectively.

The results show that the values for ultimate strength and Young’s modulus of borophene membrane in zigzag direction are smaller than that in armchair direction. This result demonstrates that the monolayer borophene is anisotropic in mechanical properties, suitable for previous reports^5,10^.

### Influence of temperature on mechanical characteristics of borophene membrane in uniaxial tension test

With the arm of studying the influence of temperature on the mechanical characteristics of borophene membrane during the uniaxial tension test, a series of MD simulations involving zigzag and armchair directions of the pristine model at various temperatures from 1 to 600 K were performed. Figures of the borophene sheet deformation and destruction, stress–strain curves, and Young’s moduli were generated for the analysis.

The fracture behavior of the borophene membrane at various temperatures in the zigzag tension is shown in Supplementary Fig. S3. The results show that when the temperature rises from 1.0 to 600 K, the crack shapes did not change significantly. Thus, the temperature does not affect spreading the crack in a direction perpendicular to the direction of tension. The borophene membranes were broken entirely when the deformation reached 16.745%, 13.277%, 11.491%, 10.033%, 8.505%, 7.476%, and 6.859%, respectively, when the temperature values changing from 1.0 to 600 K.

Supplementary Figure S4 displays the fracture processes at different temperatures under the tension along the armchair direction. Unlike the tension process along the zigzag direction, under armchair tension, it is interesting that the temperature has a significant influence on the spread of the crack. Specifically, when the temperature is low (at 1 K), the crack is mainly propagated in the direction which has an angle of 60° between the tension direction and cracks direction. When the temperature increases, the cracks will propagate perpendicular to the tension direction combined with the cracks will propagate in the direction of 60° compared to the direction of tension. As the temperature gets higher, the propagation of the crack in a direction perpendicular to the direction of tension becomes more dominant.

**Figure 4:**
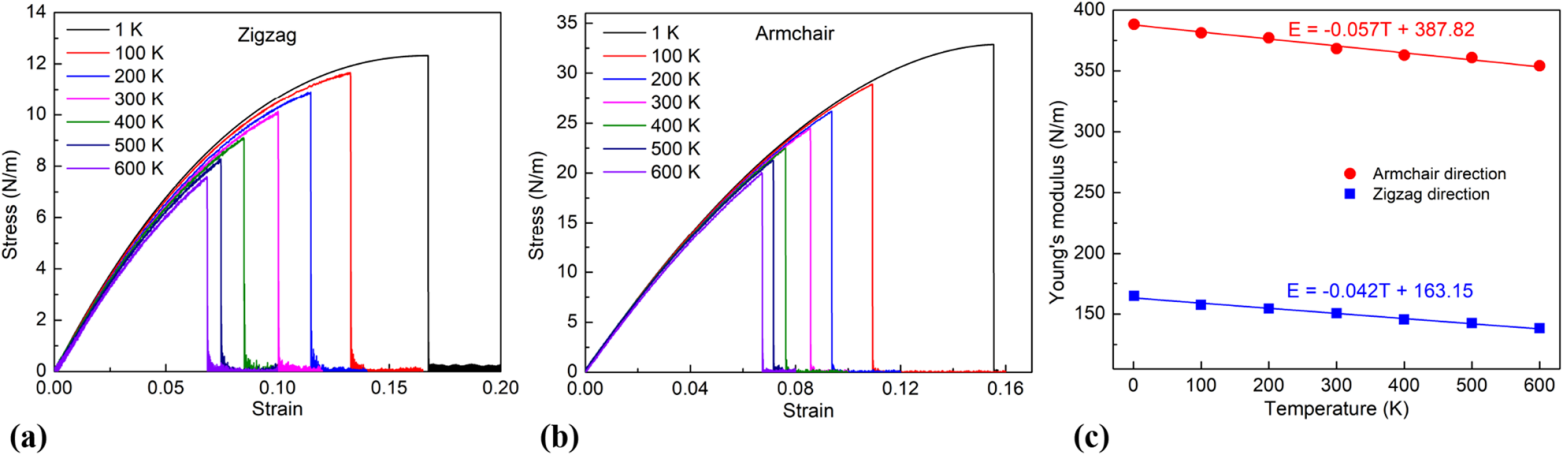
Mechanical properties of the borophene membrane at various temperatures. (**a**) Stress–strain relationship of the borophene membranes in the zigzag tension. (**b**) Stress–strain relationship of the borophene membranes in the armchair tension. (**c**) Young’s moduli of the borophene membranes in the zigzag and armchair direction under uniaxial tension.

Figure 4 displays the relationship of stress–strain, Young’s modulus of the borophene membrane at various temperatures. The results showed that temperature strongly influences the stress–strain curves. As the temperature increases, the atoms move faster, increasing thermal vibrational instabilities. Furthermore, the higher the temperature, the stronger the intensity and amplitude of the thermal fluctuations of the atoms, making the bond more susceptible to fracture. This effect results in the softening material and more vulnerability to destruction at high temperatures^40,41^. Therefore, as the temperature increases, the more ultimate strength reduces, the more likely fracture will occur. Based on the stress–strain lines, Young’s moduli at different temperatures are determined in Fig. 4c. The relation between temperature and Young’s modulus can be described by a simple linear equation:

3 $$ {\text{For armchair direction}}:E = - \;0.057T + 387.82, $$

4 $$ {\text{For zigzag direction}}:E = - \;0.042T + 163.15. $$

The considerable difference in deformation behavior, ultimate strength, Young’s modulus when tensing with different directions indicates the strong anisotropy of borophene material.

To evaluate the model and the SW potential, the comparisons of the calculated results and relevant values in previous studies are presented in Supplementary Table S1. Our results are consistent with the earlier works. Young’s modulus of pristine borophene at 1 K is 164.73 Nm^−1^ and 388.33 Nm^−1^ in zigzag and armchair directions. The ultimate tensile strength and critical strain in the zigzag direction are 12.32 Nm^−1^ and 16.74%, while in the armchair direction are 32.84 Nm^−1^ and 15.54%. The results of our research are consistent with the ab initio results in Ref.^9^, consistent with the research on MD simulation in Refs.^10,42^. These values also agree with the results in Refs.^43,44^, based on the first-principles density functional theory (DFT) calculations. It proves that this model and SW potential are suitable for studying the mechanical characteristics of the monolayer borophene. This study provides valuable knowledge about the mechanical aspects of borophene and can be a reference for further investigations.

### Strain rate sensitivity in uniaxial tension test

**Figure 5:**
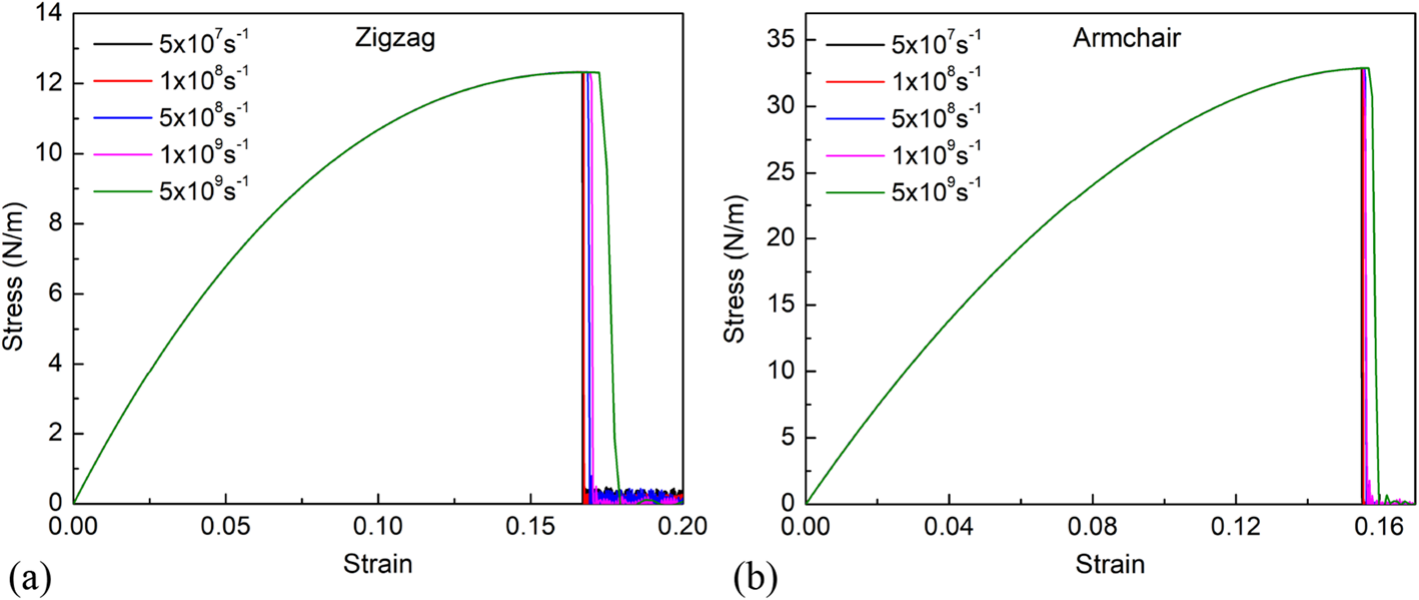
Stress–strain curves of the borophene membrane at various strain rates for uniaxial tension along (**a**) the zigzag direction, and (**b**) the armchair direction.

To study the effect of strain rates, the tension process of the pristine borophene membrane was conducted at a temperature of 1 K with different strain rates from 5 × 10^7^ to 5 × 10^9^ s^−1^, which is typically used in MD simulations. Figure 5 depicts the stress–strain curves of the borophene membrane at various strain rates for uniaxial tension along the zigzag and armchair directions. It can be seen that before the fracture occurs, the stress–strain curves do not change with different strain rates. This result indicates that Young’s modulus of borophene membrane is not affected by strain rate. The ultimate strength and fracture stress tend to increase slightly with increasing strain rate. This change can be explained as follows: at lower strain rates, response time is more, so it causes more atomic thermal fluctuations to get over the energy barrier to break their bonds and promote bond rearrangement and crack propagation^45^. As a result, the borophene membrane is more easily destroyed at low strain rates and vice versa^46^. The results show that the effect of strain rate on the mechanical properties of borophene membrane is much lower than that of other factors such as temperature and porosity.

### Influence of porosity on mechanical characteristics of borophene membrane in biaxial tension

In addition to the studies of uniaxial tension in the previous section, we study the mechanical behavior of borophene membranes under biaxial tension in this section. During biaxial tension, the loading is performed along with the two directions simultaneously at the same strain rate. Here we use the strain rate equal to the strain rate in the uniaxial tensile study.

**Figure 6:**
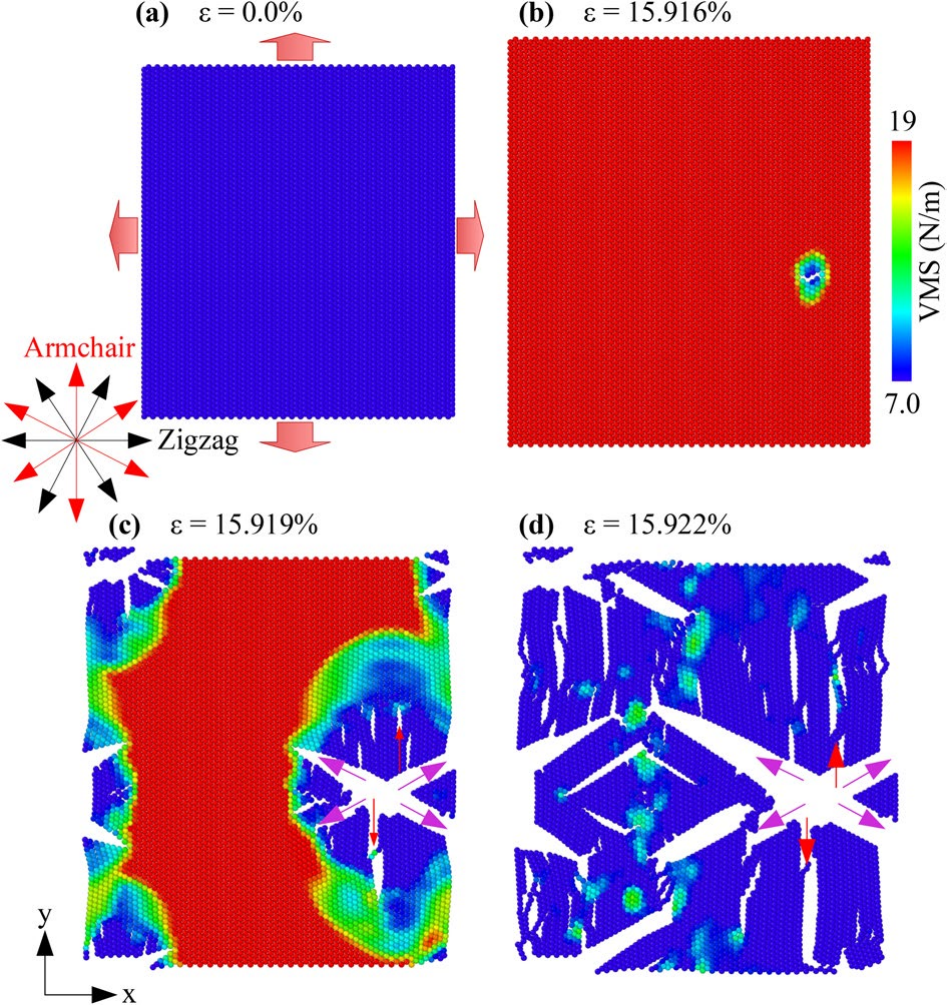
The von Mises stress distribution and structural evolution of monolayer borophene in biaxial tension at 1 K.

The deformation and fracture process of monolayer borophene (perfect model) during the biaxial tension process at 1 K are shown in Fig. 6. All atoms of the borophene nanosheet are colored by the von Mises stress (VMS) values. Here the red indicates the higher VMS, and the blue represents the lower VMS. As strain increases, the stress in the sheet increases. When the strain reaches a value of 15.916%, the sheet starts to appear cracks. These cracks spread rapidly until the sheet is completely destroyed. Compared with the process of uniaxial tension in zigzag direction (Fig. 2a) and armchair direction (Fig. 2b), the morphology of the borophene sheet when applied biaxial tensile is more rough and complex. The cracks not only spread in the direction of the red arrow but also in the pink arrow. Interestingly, the cracks spread in the armchair direction, indicating that the edge energy in the armchair edge is lower than the zigzag edge. Thus, the fracture deformation prefers to spread in this direction.

With different porosities, the von Mises stress distribution and structural evolution of monolayer borophene in biaxial tension at 1 K are exhibited in Supplementary Fig. S5. The results show that the stress will be concentrated at the pore sites under the biaxial tension process with the nanoporous membranes. The cracks will begin to appear at pore sites. Interestingly, the cracks spread in not only the armchair direction but also the zigzag direction. This phenomenon shows that the pores significantly influence the deformation behavior of the borophene membrane. In addition, the results also showed that the higher the porosity, the lower the fracture strain of the sheet.

**Figure 7:**
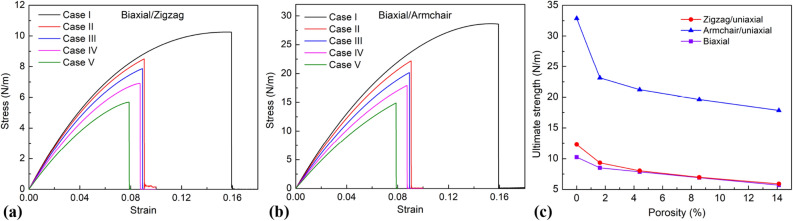
Tensile properties of the borophene membrane with various porosities. (**a**) Stress–strain curves in the zigzag direction as the borophene membranes are under biaxial tensile loading. (**b**) Stress–strain curves in the armchair direction as the borophene membranes are under biaxial tensile loading. (**c**) Ultimate strength.

Figure 7 exhibits the tensile properties of the borophene membrane with various porosity. Figure 7a shows the tress–strain curves in the zigzag direction as the borophene membranes are under biaxial tensile loading. Figure 7b shows the stress–strain curves in the armchair direction as the borophene membranes are under biaxial tension. The results show that when biaxial tension at the same strain rate, the stress in the *x-*direction (zigzag direction) is less than the stress along the *y*-direction (armchair direction). When the stress reaches the critical limit of the *x*-direction, the sheet will be destroyed simultaneously in both directions. This is shown by equal fracture strain values in the *x* and *y* directions and is also shown in Supplementary Fig. S5. The effects of porosity on the ultimate strength are shown in Fig. 7c. The results show that the porosity dramatically affects the mechanical properties of the material. As the porosity increases, the fracture strength and fracture strain decrease.

### Influence of temperature on mechanical characteristics of borophene membrane in biaxial tension

To investigate the influence of temperature on the biaxial tensile, MD simulations were performed of the biaxial tension process at a strain rate of 10^8^ s^−1^ for a temperature range from 1.0 to 600 K. The fracture behavior of borophene membrane in biaxial tension with different temperatures are shown as Supplementary Fig. S6. Similar to the uniaxial tension, as temperature increases, the fracture strain decreases. In addition, the results also show that at each different temperature value, the fracture strain under biaxial tension is smaller than the fracture strain under uniaxial tension. This proves that under biaxial tension the borophene sheet is more susceptible to destroy than uniaxial tension. At a low-temperature value (1 K), the cracks favorably spread and develop in the armchair direction and the direction with an angle 60° from the *y-*direction, which is also the armchair direction. As the temperature increases, the cracks in direction with an angle 60° from the *y-*direction decrease gradually. At high temperatures, the propagation of the crack in a direction perpendicular to the loading direction will prevail. Unlike the uniaxial tension, the fracture behavior of the borophene sheet under the biaxial tension is more complicated, and the crack appears more. This behavior proves that the sheet is loaded in both directions under the biaxial tension so that the sheet will crack more, the crack shape is rougher.

**Figure 8:**
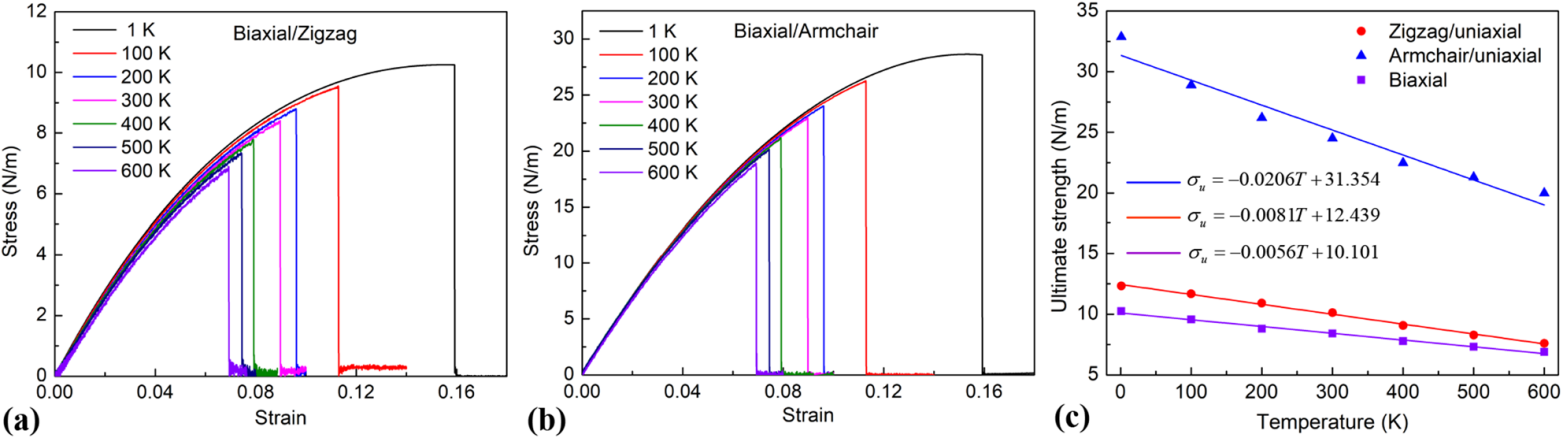
Tensile characteristics of the borophene membrane with various temperatures. (**a**) Stress–strain relationship in the zigzag direction as the borophene membranes are under biaxial tensile loading. (**b**) Stress–strain relationship in the armchair direction as the borophene membranes are under biaxial tensile loading. (**c**) Ultimate strength of the borophene membranes under uniaxial tension and biaxial tension.

Figure 8 reveals the tensile characteristics of the borophene membrane under biaxial tension with various loading temperatures. It shows that for the biaxial tension process, the ultimate strength, failure strain reduces as growing the temperature. From the stress–strain curves, we see that at different temperatures, after the borophene membrane is stretched until reaching the critical strength value, it will fracture simultaneously in both the zigzag and armchair directions. Because the stress–strain plot suddenly reduces the maximum value to the zero value at the same strain value for both directions. Figure 8c shows the ultimate strength of the borophene sheet under uniaxial tension and biaxial tension at different temperatures. The results show that when stretching the biaxial tension, the ultimate strength value is less than the ultimate strength value for uniaxial stretching. The ultimate strength values at different temperatures can be calculated using the following linear functions:

5 $$ {\text{For armchair direction }}\left( {\text{uniaxial tension}} \right):\sigma_{u} = - \;0.0206T + 31.354, $$

6 $$ {\text{For zigzag direction }}\left( {\text{uniaxial tension}} \right):\sigma_{u} = - \;0.0081T + 12.439, $$

7 $$ {\text{For biaxial tension}}:\sigma_{u} = - \;0.0056T + 10.101. $$

### Effect of temperature on thermal conductivity

We will examine the effect of borophene sheet size on thermal conductivity in this section. Previous studies have shown that the thermal conductivity is strongly influenced by the size of the sheet^47,48^. To achieve the thermal conductivity of the infinite-length for a borophene sheet, in cases of different porosity, the sheets were investigated with lengths varying from 10.3 to 60.7 nm.

**Figure 9:**
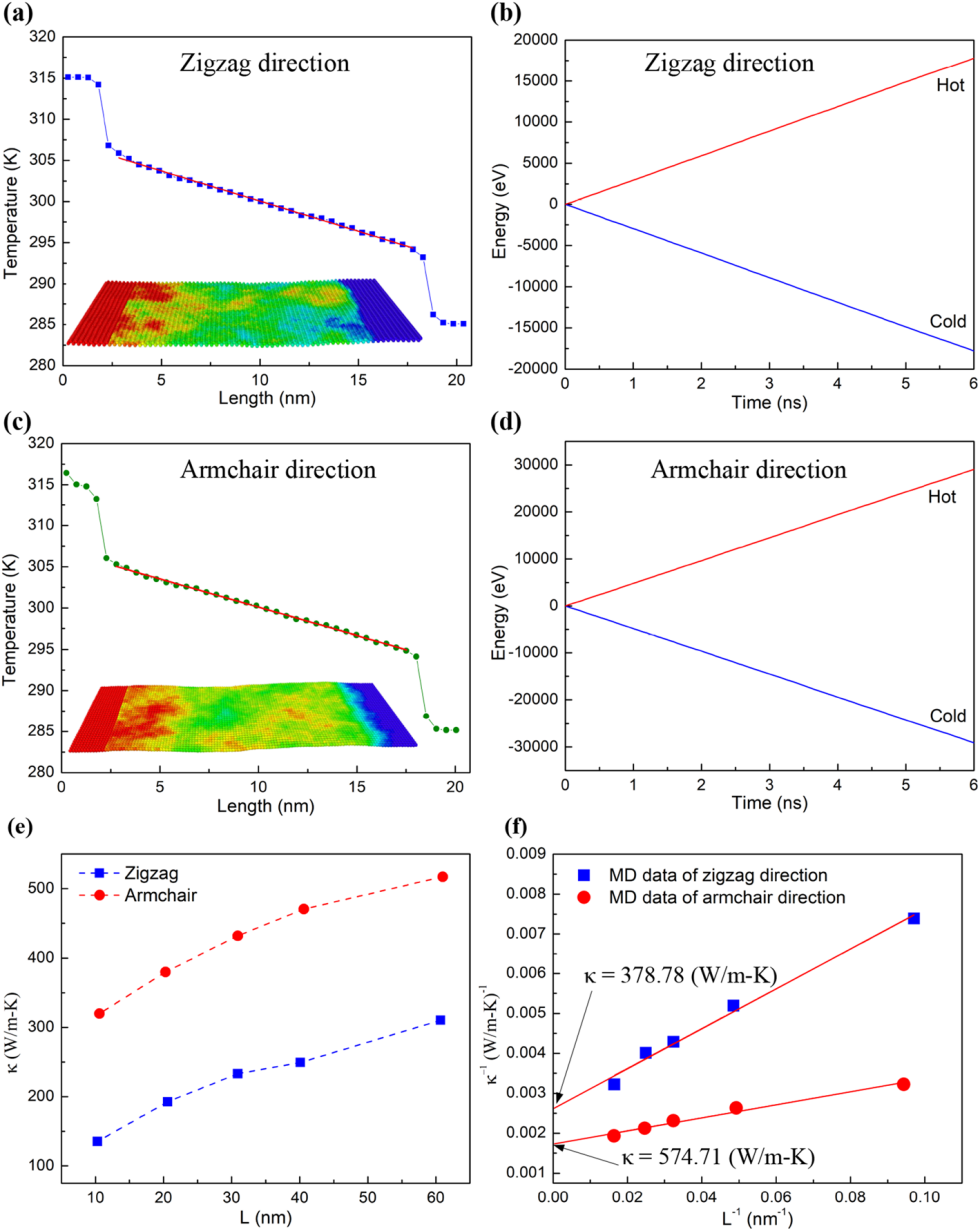
(**a**) The temperature profile in the zigzag direction of the borophene membrane at 300 K with L = 20.6 nm. (**b**) Thermal energy is subtracted from and added to the cold and hot regions respectively with respect to the time. (**c**) The temperature profile in the armchair direction of the borophene membrane at 300 K with L = 20.6 nm. (**d**) Thermal energy is subtracted from and added to the cold and hot regions respectively with respect to the time. (**e**) Dependence of thermal conductivity on the system size of monolayer borophene in zigzag and armchair directions. (**f**) Linear fitting of 1/κ and 1/L for zigzag and armchair directions.

Figure 9a–d shows an example of the stable temperature distribution for monolayer borophene at 300 K with L = 20.6 nm along with the zigzag and armchair directions. The red line in Fig. 9a,c represent the linear fitting of the temperature profile results (*dT/dx)*. Figure 9b,d reveal that the thermal energy is subtracted from the cold area and added to the hot area with respect to the time. The results show that the slope of the energy-time curves is linear, meaning that energy is subtracted from the cold area and added to the hot area at a constant rate. The results also show that the thermal energy subtracted from the cold area is equal to the energy added to the hot area, proving the total energy is conserved. From the results, we can determine the energy gradient over time (*dE/dt*). Using the formula (15) in the Method section, we can determine the thermal conductivity of borophene sheets with a length of 20.6 nm. The calculated *κ* values are 192.58 (W/m-K) and 379.93 (W/m-K), respectively, according to the zigzag and armchair directions. With lengths varying from 10.3 to 60.7 nm, simulation results are shown in Supplementary Figs. S7–S10.

Figure 9e displays the dependence of thermal conductivity on the system size of monolayer borophene in zigzag and armchair directions at 300 K. The results exhibit that the thermal conductivity is strongly influenced by the system size. With lengths ranging from 10.3 to 60.7 nm, *κ* is determined as 135.35, 192.58, 233.14, 249.41, 310.45 W/m-K in the zigzag direction and 310.16, 379.93, 432.09, 470.57, 517.21 W/m-K in the armchair direction. The longer the length sheet, the more *κ* increases. In addition, the results also show that *κ* in the zigzag direction is lower than in the armchair direction. This result indicates that the borophene membrane is anisotropic in thermal conductivity, this is in accordance with a previous study^49^. To compute the thermal conductivity of the infinite systems based on a finite amount of NEMD data, a process to inverse *κ* values according to the inverse of *L* is used for extrapolation. We use the formula of Schelling et al.^50^,

8 $$ \frac{1}{\kappa (L)} = \frac{1}{{\kappa_{\infty } }}\left( {\frac{\lambda }{L} + 1} \right), $$

where *κ*_*∞*_, *L,* and *λ* represent the intrinsic thermal conductivity of an infinite length system, the length of the system, and the effective phonon mean free path, respectively.

The linear fitting of 1*/κ* and 1*/L* for zigzag direction and armchair directions are depicted in Fig. 9f. The intrinsic thermal conductivity of borophene can be attained at the limit of 1*/L* → 0. We obtain *κ* of monolayer borophene is 378.78 W/m-K in the zigzag direction, while in armchair direction is 574.71 W/m-K. Compared with graphene material, the *κ* of borophene is lower than the *κ* of graphene with *κ* between 3000 and 5000 W/m-K^51^. Another 2D material with a honeycomb atomic structure is h-BN, with *κ* = 277.78 W/m-K for armchair direction and *κ* = 588.24 W/m-K for zigzag direction^52^.

**Figure 10:**
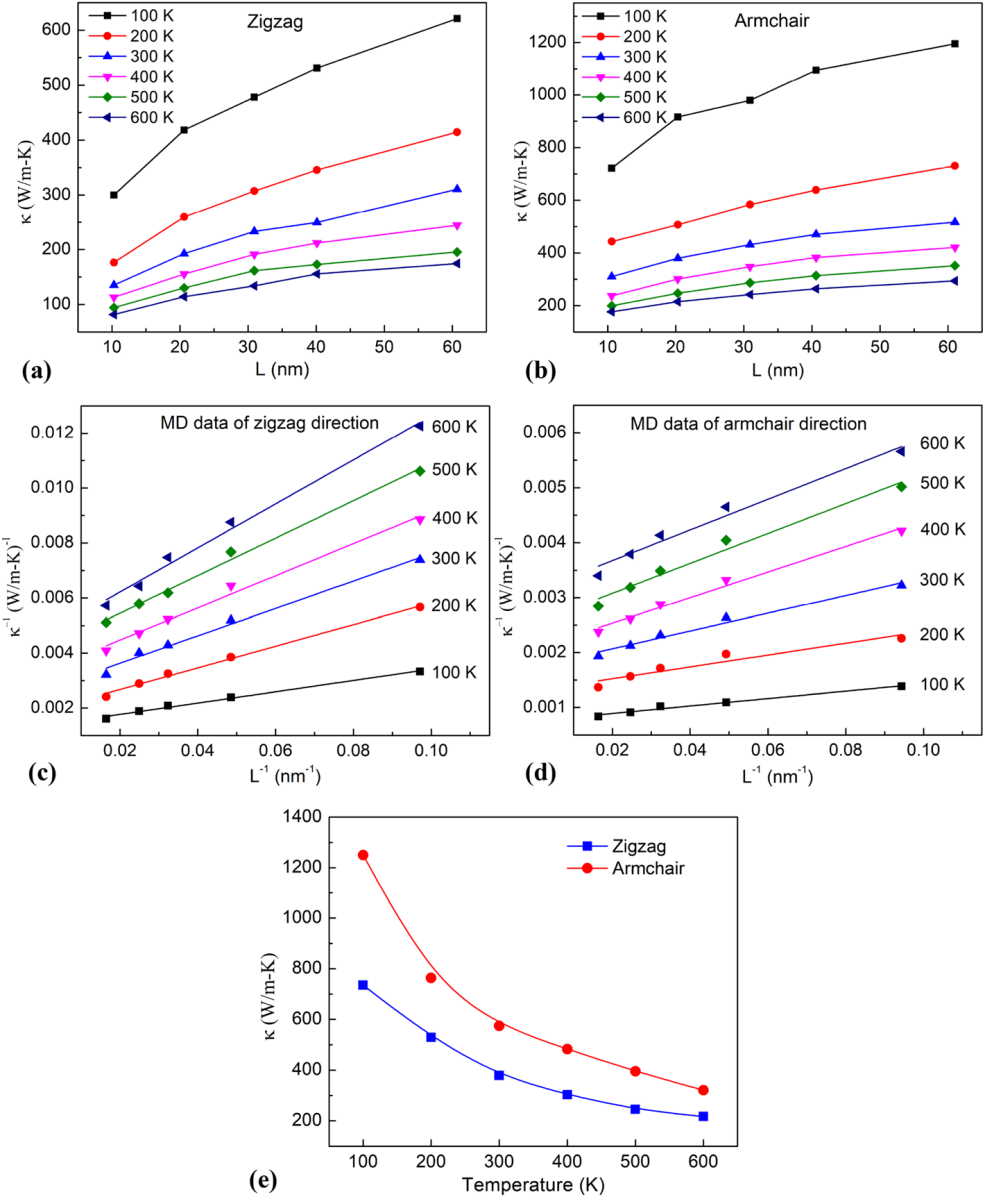
(**a,b**) Dependence of thermal conductivity on the system size of monolayer borophene in zigzag and armchair directions with different temperatures. (**c,d**) Linear fitting of 1/κ and 1/L for zigzag and armchair directions. (**e**) The intrinsic thermal conductivity of monolayer borophene at various environment temperatures.

The environment temperature greatly affects the thermal conductivity of the material. Therefore, it is necessary to understand the influence of temperature on thermal conductivity. The *κ* values calculated at different environment temperatures by different lengths are shown in Fig. 10a,b corresponding to the zigzag and armchair directions. The results show that as the temperature gets higher, the thermal conductivity decreases. This phenomenon can be explained by previous studies to be due to Umklapp phonon–phonon scattering^53^. High temperatures result in the population of phonons with great momenta is grown. When these phonons with large momentum collided, their sum might point outside the first Brillouin zone. Therefore, with increasing the temperature, the Umklapp scattering increase. The more Umklapp scattering, the more limiting the heat transfer in the material. As a result, as increasing temperature, the thermal conductivity decreases. This result is in accordance with Park et al. report^54^, which studied the thermal transport in thorium dioxide. This result is also reasonable in agreement with Islam et al.^55^ for 2D silicon carbide.

To determine the intrinsic thermal conductivity of a material at different ambient temperatures, an extrapolation process from the inverse *κ* values according to the inverse of *L* is performed, as shown in Fig. 10c,d. From the extrapolation value when 1*/L* → 0, we can determine intrinsic thermal conductivity values under different environmental temperatures, as shown in Fig. 10e. In general, the higher the ambient temperature increases, the more *κ* is reduced, which is consistent with previous studies^54,55^. In addition, the results showed that *κ* in the zigzag direction is lower than *κ* in the armchair direction. This result proves that borophene sheet material is anisotropic in heat transfer, consistent with the previous work^49^. In the previous first-principle study of the lattice thermal conductivity of borophene by Sun et al.^49^, the group velocities and phonon lifetimes of borophene along the zigzag direction are significantly lower than the armchair direction. It leads to the thermal conductivity of borophene is lower in the zigzag direction. This result was also reported by Mortazavi et al.^29^ by calculating the phonon density of stated (DOS) for borophene. The difference in group velocities reported by Sun et al., along with DOS analysis by Mortazavi et al., provides a consistent explanation for the anisotropy in thermal conductivity of borophene.

### Effect of porosity on thermal conductivity

**Figure 11:**
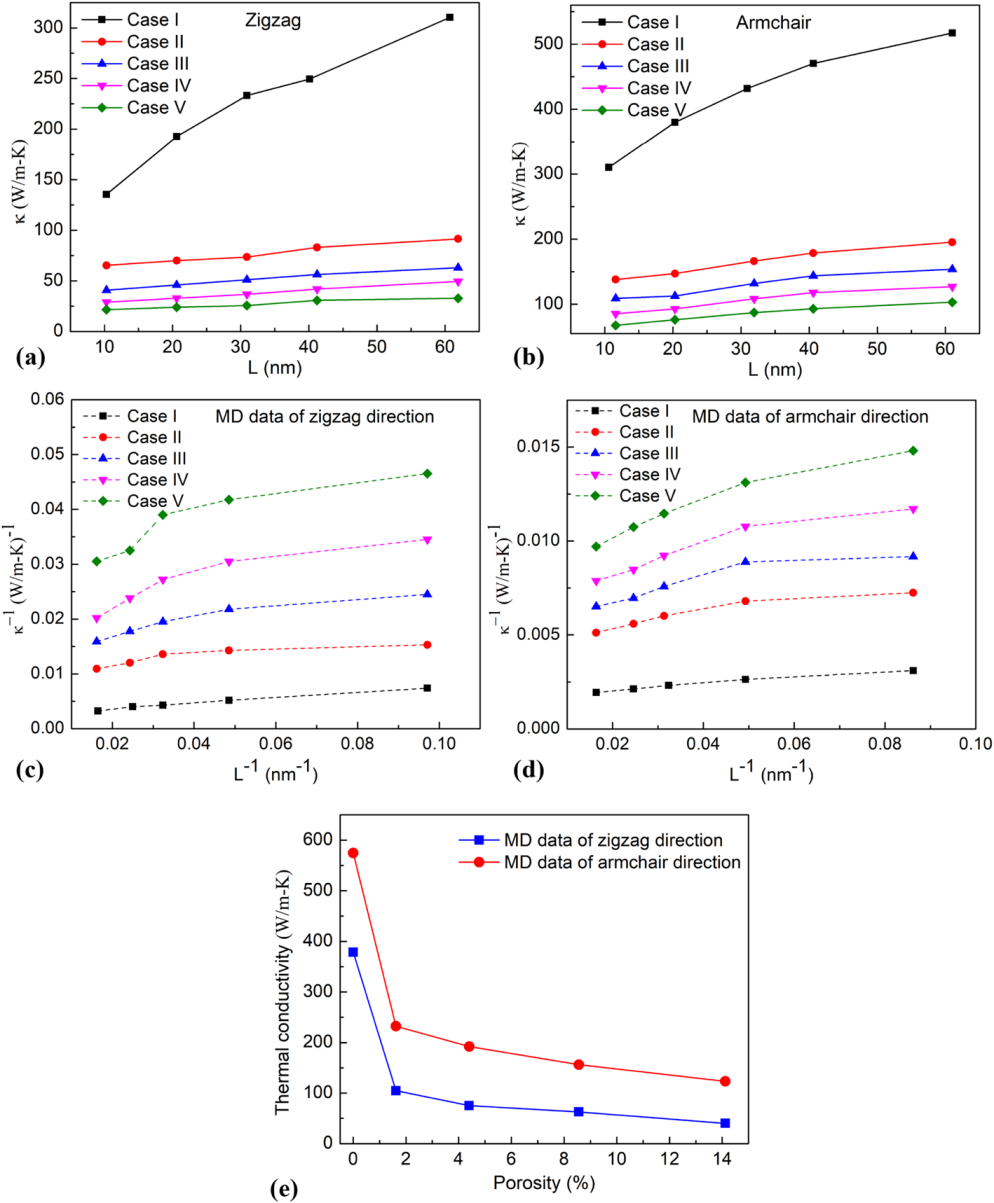
(**a,b**) Dependence of thermal conductivity on the system size of monolayer borophene in zigzag direction and armchair direction with different porosities. (**c,d**) Relationship between 1/κ and 1/L for zigzag and armchair directions. (**e**) The intrinsic thermal conductivity of monolayer bororphene with different porosities.

Supplementary Figure S11 shows the temperature profile and temperature distribution of the monolayer borophene with a porosity of 4.40% for the zigzag and armchair directions at 300 K. The results reveal that the defects affect the temperature distribution, the sudden drop in temperature at the vacancy defects. The vacancies defect or porosity is expected to contribute to the thermal resistance of the nanosheet, resulting in reduced thermal conductivity, as shown in Fig. 11. As revealed in Fig. 11, as the porosity grows for each equal length, the samples’ thermal conductivity reduces. From the MD simulation data at different length values, we can extrapolate the *κ* values of the infinite length system, as shown in Fig. 11e.

Figure 11e shows that the greater the porosity density, the lower the thermal conductivity. Based on the classical lattice thermal conductivity theory^56,57^, the *κ* of defective material satisfies the relation

9 $$ \frac{1}{\kappa } \propto \frac{1}{{l_{{phonon{-}phonon}} }} + \frac{1}{{l_{{phonon-defect}} }}, $$

where *l*_*phonon-phonon*_ is the phonon–phonon scattering length, and *l*_*phonon-defect*_ is the phonon-defect scattering length.

According to previous studies^47,58^, as the defect density increases, the phonon-defect scattering increases and the *l*_*phonon-defect*_ decreases. As a result, according to Eq. (9) we can see that the *κ* of borophene nanosheet decreases with increasing porosity due to the phonon-defect scattering effect, which is in good agreement with our previous study^58^. Moreover, these MD simulation results are in accordance with the earlier works^59–62^.

## Discussions

In summary, we explored the influences of porosity and temperature on the structural evolution and mechanical characteristics of borophene membranes in uniaxial tension and biaxial tension. The borophene membranes with different porosities of 0.0%, 1.62%, 4.40%, 8.56%, and 14.12% were used in the study. The results showed that the mechanical characteristics of borophene membranes, including Young’s modulus, ultimate strength decreased as porosity increased. The defects or porosity negatively affect the mechanical properties of the membranes. Under the tension process, the stress concentrates around the vacancy site and causes the crack to start appearing at the vacancy sites. Consequently, increasing the porosity leads to Young’s modulus and ultimate strength significantly decrease. The borophene membrane has anisotropic properties and its Young’s modulus and ultimate strength in the armchair direction higher than that in the zigzag direction.

The effects of porosity and temperature on thermal conductivity were also explored. The borophene membrane is anisotropy in thermal conductivity. The thermal conductivity in the zigzag direction is lower than that in the armchair direction. The porosity also affects thermal conductivity significantly. The greater the porosity, the lower the thermal conductivity caused by the defect scattering. In addition, the results also show that as the temperature grows, the thermal conductivity reduces.

## Methods

### MD simulations for tension process

To eliminate the boundary effect of the system, periodic boundary conditions are incorporated in three directions. Besides, in the *z-*direction, a 5.0 nm vacuum gap is placed below and above the borophene sheet to eliminate interactions of adjacent sheets. To represent atomic interactions in borophene sheets, a Stillinger–Weber (SW) potential suggested by Jiang et al.^42^ is employed. This potential is effective in molecular dynamics simulation for borophene sheets and has been widely used in previous studies^63,64^. All simulations in this study were performed by LAMMPS software^65^. To display and analyze the simulation results, we use OVITO software^66^.

In this study, the mechanical properties of borophene sheets were studied through uniaxial and biaxial tensile tests with different temperatures and different porosities. Before the tensile process, the borophene sheets are energy-minimized structures using the conjugate gradient (CG) algorithm to obtain an equilibrium system. Then, the system is then relaxed at a predetermined temperature (i.e., 1.0 K, 100.0 K, 200.0 K, 300.0 K, 400.0 K, 500.0 K, and 600.0 K) using an NPT ensemble. To release internal stress, a relaxing process is performed within 250 ps with a time step of 0.0005 ps, at atmospheric pressure. Then, the uniaxial tension test along with the zigzag, armchair directions, and biaxial tension test are performed with a constant engineering strain rate of 10^8^ s^−1^. All tension processes are performed in the NPT ensemble with the time step 0.0005 ps. Engineering strain is defined by the following formula:

10 $$\varepsilon =\frac{L-{L}_{0}}{{L}_{0}},$$

where, *L*_0_ and *L* are the lengths of the sample before the deformation, and the instantaneous length, respectively.

The stress component is given by Virial theorem^67^

11 $$ \sigma = \frac{1}{V}\sum\limits_{a \in V} {\left[ { - m_{a} v_{a} \otimes v_{a} + \frac{1}{2}\sum\limits_{a \ne b} {(r_{ab} \otimes F_{ab} )} } \right]} , $$

where, *m*_*a*_ and *v*_*a*_ are the mass and the velocity vector of the atom *a*. The symbol $\otimes$ denotes the Tensor product, *r*_*ab*_ denotes the distance vector of the atom *a* with respect to atom *b*. *F*_*ab*_ is the force vector on atom *a* caused by atom *b*. *V* is defined *V* = *A *$\times$* t*, here *V* represents the volume of the structure, *A* is the surface area, and *t* is the membrane nominal thickness. Like previous studies with 2D materials^68,69^, the length along the out-of-plane direction of the simulation box varies during the tensile test. Then, the membrane nominal thickness *t* cannot be easily determined, so this study also used the N/m unit for the stress values, Young's modulus of the borophene membrane.

The von Mises stress *σ*_*von*_ is determined as:

12 $$ \sigma_{von}^{2} = \frac{1}{2}\left[ {\left( {\sigma_{xx} - \sigma_{yy} } \right)^{2} + \left( {\sigma_{yy} - \sigma_{zz} } \right)^{2} + \left( {\sigma_{zz} - \sigma_{xx} } \right)^{2} + 6\left( {\sigma_{xy}^{2} + \sigma_{yz}^{2} + \sigma_{zx}^{2} } \right)} \right]. $$

### Thermal conductivity calculations

Before performing the heat flux process, the system is relaxed at a given temperature by NVT and NVE methods respectively in 500 ps intervals, with a time step of 0.0005 ps. Following the equilibrium process, the thermal conductivity of the borophene sheet will be evaluated using the NEMD method. The calculated model is divided into 40 slabs along the length of the plate. Hot and cold zones are defined as shown in Fig. 1b. Atoms in the hot and cold regions were respectively set at *T*_*h*_ = *T*(1 + Δ) K and *T*_*c*_ = *T*(1 − Δ) K by Langevin thermostat, where Δ = 0.05, and *T* is the specific temperature (100, 200, 300, 400, 500, 600 K). The simulation process to calculate the thermal conductivity is 6.0 ns. During this process, to keep the cold and hot regions at a prescribed temperature, energy is continuously removed from the cold region and added to the hot zone. After a while, the temperature profile will reach a steady state. The temperature of each slab is determined as follows^70,71^:

13 $$ T_{k} = \frac{1}{{3n_{k} k_{B} }}\sum\limits_{i \in k}^{{n_{k} }} {m_{i} v_{i}^{2} } , $$

where, *n*_*k*_ denotes the number of atoms in *k*th slab, *k*_*B*_ is Boltzmann’s constant, *m*_*i*_ and *v*_*i*_ are the atomic mass and velocity of atom *i*.

Heat flux is determined as follows^72^:

14 $$ J = \frac{dE/dt}{A}, $$

where *A* is the cross-section area, its assuming nominal thickness of borophene sheet is 0.29 nm, *dE/dt* stand for the time rate of change of the total amount of accumulated energy.

After a transient period, the temperature gradient is attained (*dT/dx*). Then, the thermal conductivity is calculated using the Fourier’s heat condition law:

15 $$ k = \frac{J}{dT/dx}. $$

## Supplementary Information

Supplementary Figure S1.

Supplementary Figure S2.

Supplementary Figure S3.

Supplementary Figure S4.

Supplementary Figure S5.

Supplementary Figure S6.

Supplementary Figure S7.

Supplementary Figure S8.

Supplementary Figure S9.

Supplementary Figure S10.

Supplementary Figure S11.

Supplementary Table S1.
