# Supplementary material for: Understanding porosity and temperature induced variabilities in interface, mechanical characteristics and thermal conductivity of borophene membranes
Source: Sci Rep. 2021 Jun 9;11:12123. doi: 10.1038/s41598-021-91705-2 (PMC8190318; doi:10.1038/s41598-021-91705-2)
Supplement: Supplementary file 2 — Supplementary Figure S2. [file 41598_2021_91705_MOESM2_ESM.docx]

**Supplementary Figure 2.** Deformation and fracture process of monolayer borophene in armchair tension at 1 K with various porosities.
