# Supplementary material for: Understanding porosity and temperature induced variabilities in interface, mechanical characteristics and thermal conductivity of borophene membranes
Source: Sci Rep. 2021 Jun 9;11:12123. doi: 10.1038/s41598-021-91705-2 (PMC8190318; doi:10.1038/s41598-021-91705-2)
Supplement: Supplementary file 6 — Supplementary Figure S6. [file 41598_2021_91705_MOESM6_ESM.docx]

**Supplementary Figure 6.** Fracture behavior of borophene membrane in biaxial tension with different temperatures.
