# Supplementary material for: Understanding porosity and temperature induced variabilities in interface, mechanical characteristics and thermal conductivity of borophene membranes
Source: Sci Rep. 2021 Jun 9;11:12123. doi: 10.1038/s41598-021-91705-2 (PMC8190318; doi:10.1038/s41598-021-91705-2)
Supplement: Supplementary file 10 — Supplementary Figure S10. [file 41598_2021_91705_MOESM10_ESM.docx]

**Supplementary Figure 10.** (**a**) The temperature profile in the zigzag direction of the borophene membrane at 300 K with L = 60.7 nm. (**b**) Thermal energy is subtracted from and added to the cold and hot regions respectively with respect to the time. (**c**) The temperature profile in the armchair direction of the borophene membrane at 300 K with L = 60.7 nm. (**d**) Thermal energy is subtracted from and added to the cold and hot regions respectively with respect to the time.
