# Supplementary material for: Understanding porosity and temperature induced variabilities in interface, mechanical characteristics and thermal conductivity of borophene membranes
Source: Sci Rep. 2021 Jun 9;11:12123. doi: 10.1038/s41598-021-91705-2 (PMC8190318; doi:10.1038/s41598-021-91705-2)
Supplement: Supplementary file 11 — Supplementary Figure S11. [file 41598_2021_91705_MOESM11_ESM.docx]

**Supplementary Figure 11.** Temperature profile in borophene nanosheet with porosity of 4.40% along the zigzag and armchair directions at 300 K and L = 20 nm.
