# Supplementary material for: Understanding porosity and temperature induced variabilities in interface, mechanical characteristics and thermal conductivity of borophene membranes
Source: Sci Rep. 2021 Jun 9;11:12123. doi: 10.1038/s41598-021-91705-2 (PMC8190318; doi:10.1038/s41598-021-91705-2)
Supplement: Supplementary file 12 — Supplementary Table S1. [file 41598_2021_91705_MOESM12_ESM.docx]

**Supplementary Table 1**

Comparison of the calculated results and relevant values obtained by previous works

| Material | Young’s modulus  (N/m) | Fracture strength (N/m) | Failure strain (%) | Temperature | References |
| --- | --- | --- | --- | --- | --- |
| Borophene | 170 (zigzag)  398 (armchair) | -  - | -  - | 0 K | Mannix et al. [9] |
| Borophene | 163 (zigzag)  394 (armchair) | 13.2  24.1 | 15.3  10.2 | 1 K | Zhou et al. [10] |
| Borophene | 162.7 (zigzag)  385.0 (armchair) | 12.3  32.9 | 17.0  16.0 | 1 K | Jiang  et al. [43] |
| Borophene | 158 (zigzag)  397 (armchair) | 12.4  25.2 | 16.0  14.0 | 0 K | Zhong et al. [44] |
| Borophene | 166 (zigzag)  389 (armchair) | 12.98  24.0 | 15.0  10.0 | 0 K | Wang et al. [45] |
| Borophene | 164.73 (zigzag)  388.33 (armchair) | 12.32  32.87 | 16.740  15.541 | 1 K  1 K | This research |
